# Cytoskeletal Protein Translation and Expression in the Rat Brain Are Stressor-Dependent and Region-Specific

**DOI:** 10.1371/journal.pone.0073504

**Published:** 2013-10-04

**Authors:** Petra Sántha, Magdolna Pákáski, Eszter K. Fodor, Örsike Cs Fazekas, Sára Kálmán, János Kálmán, Zoltán Janka, Gyula Szabó, János Kálmán

**Affiliations:** 1 Alzheimer's Disease Research Centre, Department of Psychiatry, University of Szeged, Szeged, Hungary; 2 Department of Psychiatry, University of Szeged, Szeged, Hungary; 3 Department of Pathophysiology, University of Szeged, Szeged, Hungary; Alexander Fleming Biomedical Sciences Research Center, Greece

## Abstract

Stress is an integral component of life that can sometimes cause a critical overload, depending on the qualitative and quantitative natures of the stressors. The involvement of actin, the predominant component of dendritic integrity, is a plausible candidate factor in stress-induced neuronal cytoskeletal changes. The major aim of this study was to compare the effects of three different stress conditions on the transcription and translation of actin-related cytoskeletal genes in the rat brain. Male Wistar rats were exposed to one or other of the frequently used models of physical stress, i.e. electric foot shock stress (EFSS), forced swimming stress (FSS), or psychosocial stress (PSS) for periods of 3, 7, 14, or 21 days. The relative mRNA and protein expressions of β-actin, cofilin and mitogen-activated protein kinase 1 (MAPK-1) were determined by qRT- PCR and western blotting from hippocampus and frontal cortex samples. Stressor-specific alterations in both β-actin and cofilin expression levels were seen after stress. These alterations were most pronounced in response to EFSS, and exhibited a U-shaped time course. FSS led to a significant β-actin mRNA expression elevation in the hippocampus and the frontal cortex after 3 and 7 days, respectively, without any subsequent change. PSS did not cause any change in β-actin or cofilin mRNA or protein expression in the examined brain regions. EFSS, FSS and PSS had no effect on the expression of MAPK-1 mRNA at any tested time point. These findings indicate a very delicate, stress type-dependent regulation of neuronal cytoskeletal components in the rat hippocampus and frontal cortex.

## Introduction

Organisms are often exposed to periods of stress throughout their whole lives; most of these episodes can be controlled and may even be necessary for survival. Stressful stimuli can play a relevant role as environmental factors in psychiatric disorders, such as anxiety, affective disorders and Alzheimer's disease (AD).

Recent papers implicate that stress has profound effects on the reorganization of dendritic spines of the hippocampus and the reduction of synaptic plasticity [1]–[3]. Dynamic actin cytoskeleton has a unique stress response and it mediates cellular events that underlie changes in synaptic transmission and morphology [4]–[6]. Proteomic and genomic investigations have demonstrated that cytoskeletal proteins are involved in the neurobiological processes related to stress [7], [8]. Filamentous actin (F-actin) is the major cytoskeletal component of the dendritic spines and plays a key role in the morphogenesis, maintenance and plasticity of these spines [6], [7], [9]. The actin filament dynamics are regulated by several types of proteins [10]. One of the most important is cofilin, which is regulated by the ratio of its concentration to those of actin and other actin-binding proteins [11], [12]. Another regulatory factor is the mitogen-activated protein kinase 1 (MAPK-1) which contributes F-actin stabilization and arrangement [13]. MAPK-1 is also responsible for the hyperphosphorylation of tau, leading to microtubule degeneration and cell death in AD [14].

Although β-actin is considered an internal standard gene, recent studies clearly revealed changes in β-actin transcription and translation and imbalanced functioning of the actin-regulator machinery in experimental stress models [8], [15]. This cytoskeletal remodeling results in a synaptic dysfunction, which is indicated by different forms of behavioural, cognitive and affective impairments in humans [7]. Further evidence of the involvement of cytoskeletal modification in depressive disorders has emerged from investigations of the response to antidepressant treatment [16]–[18].

Stressful stimuli lead to a variety of changes in the function, shape and proliferative capacity of brain cells [19], [20]. Previous studies have proven that acute and chronic restraint stress (RS) and electric foot shock stress (EFSS) can cause decreases in neurogenesis [21]–[23], while chronic psychosocial stress (PSS) partially blocks the early long-term potentiation of the CA1 area of the hippocampus [24]. RS can change the status of the microtubular dynamics in the rat hippocampus, causing an involution of structural neuronal plasticity, thereby playing a part in the pathophysiology of stress-related conditions [25]. As we have demonstrated in our recent study, RS induced biphasic dynamic changes in the transcription and protein translation of the main cytoskeletal component, β-actin, and its regulatory proteins, cofilin and MAPK-1, in an *in vivo* rat model, selectively in the hippocampal region [15].

It has been suggested that the effects of stress are influenced by many factors, including its type or duration, gender, age, individual sensitivity and the brain region [26]. The importance of the various parameters can be established from separate experiments. Relatively few comparative investigations have been made concerning the influence of gender, the duration of stress and the affected brain region [22], [23], [27]–[29]. In contrast to the wide methodological repertoire of available animal stress models, the cytoskeletal effects of different physical and psychological stressors have not yet been compared.

As a follow-up study to our previous work on RS, in the present study, we investigated the effects of three widely-used experimental stressors, EFSS, forced swimming stress (FSS) and PSS on mRNA and protein expression of β-actin and cofilin in rat hippocampus and frontal cortex, regions most sensitive to stress-related changes [15], [22], [27]. Furthermore, the mRNA expression of MAPK-1, a regulator of cytoskeletal components also implicated in stress was examined in these different stress modalities. The acute and chronic effects of these physical and psychological stressors were also compared.

## Materials and Methods

### Animals

Adult male Wistar rats (200–300 g; n = 6–10/group) were housed in a temperature (22±1°C) and humidity (55±5%) controlled room on a 12 h light–dark cycle (lights on from 8.30 a.m. to 8.30 p.m.) and allowed free access to tap water and rat chow. All animal procedures were approved by the Ethical Committee for the Protection of Animals in Research of the University of Szeged (approval number: I-74-4/2011.MÁB). In each of the stress procedures (EFSS, FSS and PSS), the animals were divided into 5 experimental groups. Group 1 comprised the controls, while groups 2, 3, 4 and 5 were subjected to the given stress for 3, 7, 14 or 21 days, respectively. The animals were housed 3 per cage in EFSS and FSS, and 5 per cage in PSS. The control animals were left completely undisturbed. Since each stress protocol was done as a separate experiment, each stress model had its own control group.

The day after the last stress procedures (at 8 a.m.), the rats were anaesthetized with 8% chloral-hydrate and, following the transcardial perfusion with cold saline solution, the cerebral hemispheres were separated and the hippocampus and frontal cortex were dissected on an ice-cold tile. The same animals were used to measure mRNA and protein levels, but they were selected randomly to eliminate the changes induced by laterality. The samples were frozen with dry ice powder and stored at −80°C until further experimental processing.

### Stress procedures

#### Electric foot-shock stress

EFSS was applied as in the protocol described by Tsukada et al. (2003) and Robbins and Ness (2008) by exposing the rat's footpad to a constant current produced with a foot-shock generator. In the acute stress experiment, a total of 6 random shocks, each with an intensity of 1 mA for 750 ms, were administered within a period of 2 min, daily, for 3 consecutive days. In the chronic stress experiment, 10 random shocks, 0.6 mA in intensity, lasting for 2 s were administered daily within a period of 5 min for 7, 14 or 21 consecutive days [30], [31].

#### Forced swimming stress

The FSS protocol described by Porsolt et al. (1978) was used in our experiments. Each rat was placed into a vertical Plexiglas cylinder (height 45 cm, diameter 19.4 cm) containing 32 cm of water maintained at 23°C for 10 min, then removed and allowed to dry before being returned to their cages. The water was so deep that the tails of the swimming or floating animals did not touch the bottom. The water was changed after each animal. Three identical cylinders were used, separated by opaque screens, for simultaneous testing [32]–[35].

#### Psychosocial stress

The protocol of Gerges et al. (2001) was used in our experiments. Rats were kept with the same cage mates for at least 1 week to allow the establishment of social hierarchy. At the end of that period, 2 rats from each cage, randomly chosen, were switched once a day at the same time of day from one cage to the other for a period of 3 days. Analogous procedures were carried out for periods of 7, 14 and 21 days [24], [35], [36].

### Total RNA isolation and reverse transcription

Total cellular RNA was extracted from the frontal hippocampus and frontal cortex by means of the NucleoSpin RNA II Total RNA isolation kit (Macherey-Nagel, Düren, Germany), according to the manufacturer's instructions. 0.3 µg of RNase inhibitor 40 U/µl (Fermentas, Glen Burnie, Maryland, USA) was added and the eluted RNA was stored at −80°C until further use.

Reverse transcription reactions were carried out for each RNA sample, subsequently followed by first-strand cDNA synthesis from total RNA samples by using the High Capacity cDNA Reverse Transcription Kit (Applied Biosystems, Foster City, CA, USA). 2 ng of total mRNA were transcribed into cDNA. Each reaction tube, with a total volume of 30 µl, contained 2 ng of total RNA in a volume of 15 µl, and 15 µl of transcription mix (3 µl of reverse transcription buffer, 1.2 µl (100 mM) of dNTP mix, 3 µl of random primers, 1.5 µl of Multi Scribe™ reverse transcriptase, 0.75 µl (20 U) of RNase inhibitor and RNA Free Water (Ambion, Austin, TX, USA)). The thermal cycling consisted of three cycles: the first at 25°C for 10 min, the second at 37°C for 120 min, and the final one at 85°C for 5 s. The samples were then cooled down to 4°C, and finally stored at −20°C until qRT-PCR.

### Real-time polymerase chain reaction

Reactions were performed with a RotorGene 3000 (Corbett Research, Sydney, Australia). Gene-specific primers designed by using Primer Express software (Applied Biosystems, Foster City, CA, USA) were used. The primer sequences were as follows: β-actin (forward): CCC GCG GAG TAC AAC CTT CT, (reverse): CGT CAT CCA TGG CGA ACT; cofilin (forward): GGC GGC TCT GTT CTT CTG T, (reverse): CTC CAT CAG AGA CAG CCA CA; GAPDH (forward): AGA TCC ACA ACG GAT ACA TT and (reverse): TCC CTC AAG ATT GTC AGC AA; MAPK-1 (forward): CCA AGC TCA ACC GTC TCA TC, (reverse): GGC TGG TAG GGT AGT TGA TG.

30 µl of cDNA solution was diluted with 510 µl of DNase and RNase-free water. Q-PCR was carried out in a final volume of 20 µl containing 10 µl of SYBR Green MasterMix (Roche, Basel, Switzerland), 0.5 µl of forward primer, 0.5 µl of reverse primer, and 9 µl of template cDNAs. The protocol comprised denaturation for 25 s at 95°C, annealing for 25 s at 60°C, and extension for 15 s at 72°C. The relative gene expression was normalized to glyceraldehyde-3-phosphate dehydrogenase. The results were analysed by the 2^−ΔΔCT^ method [37].

### Western blotting

The brain regions were homogenized in a solution containing 50 mM Tris buffer (pH 7.5), 150 mM NaCl, 0.1% Nonidet-P-40, 0.1% cholic acid, 2 µg/ml leupeptin, 2 mM PMSF, 1 µg/ml pepstatin and 2 mM EDTA. The homogenates were centrifuged at 10 000 g for 15 min at 4°C. The supernatants were used for protein assays. Proteins were measured with bicinchoninic acid [38].

After denaturation, 20 µg of protein were separated on 12% SDS-polyacrylamide gel and electroblotted onto nitrocellulose membranes. The samples were blocked in a solution of 0.1 M Tris-buffered saline containing 0.02% Tween 20 (TBST) supplemented with 5% non-fat milk for 1 h. The membranes were then incubated overnight with mouse monoclonal anti-β-actin (Santa Cruz, CA, USA, 1∶2000), rabbit polyclonal cofilin (D59) antibody (Cell Signaling Technology, MA, USA 1∶1000) and mouse monoclonal anti-GAPDH (Millipore, MA, USA 1∶4000). The next day, after five washes with TBST, horseradish-peroxidase-labelled anti-mouse IgG (Jackson Immunoresearch, West Grove, PA, USA 1∶1000) and horseradish-peroxidase-labelled anti-rabbit IgG (Jackson Immunoresearch, West Grove, PA, USA 1∶1000) secondary antibodies were applied for 1 h. The nitrocellulose membranes were subsequently washed five times with TBST, and then incubated with the Supersignal® West Pico Chemiluminescent Substrate (Pierce, Rockford, IL, USA) and exposed to Kodak autography film. The optical densities of the immunoreactive bands were quantified by means of Scion Image Software. The amounts of examined proteins were calculated by comparison with the optical density of internal control. For each blot of β-actin and cofilin, the relative protein level was calculated from the ratio of absorbance of β-actin/GAPDH and the ratio of the absorbance of cofilin/GAPDH. This was considered as 100% in the control group and the data of different time points were compared to this ratio.

### Statistical analysis

All data are reported as mean ± SEM; they were analyzed by two-way ANOVA with SPSS 15.0 Software: Stress types (EFSS, FSS, PSS) x Exposure times (3, 7, 14 and 21 days). Significant main effects and interactions were followed by *post hoc* comparisons using the General Linear Model. The comparison within the same groups was assessed by Student's t-test and by one-way ANOVA followed by the Bonferroni and Games-Howell *post hoc* tests; the level of significance of comparisons was taken as *p*<0.05.

## Results

### Body, adrenal gland and thymus weights of the stressed animals

**Figure 1:**
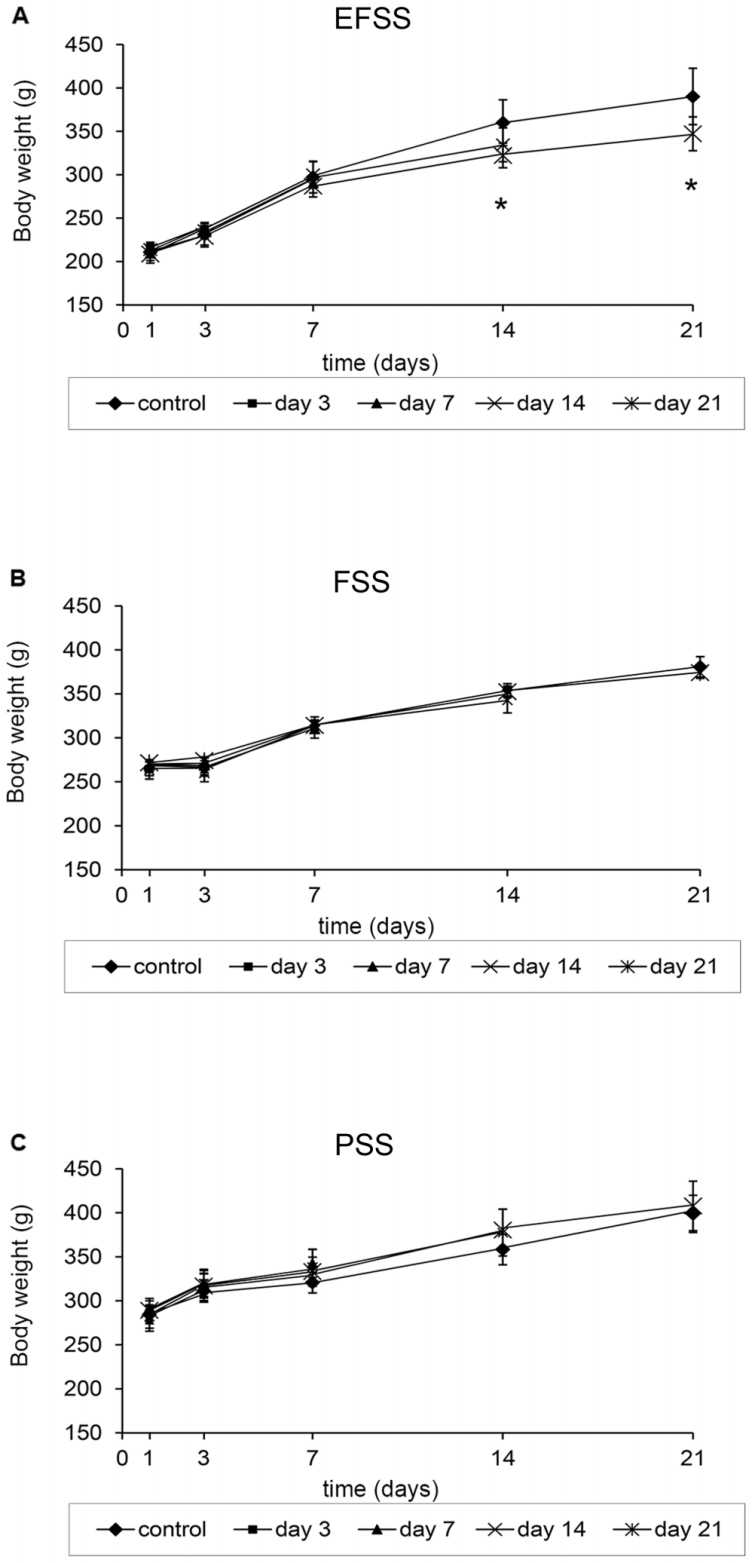
Stress type-dependent body weight alterations. Effects of electric foot shock stress (EFSS) (**A**), forced swimming stress (FSS) (**B**) and psychosocial stress (PSS) (**C**) on the overall body weight of rats, measured on days 3, 7, 14 and 21. Values for each group are means ± SEM, n = 6–10. *p<0.05 and **p<0.01: significant differences as compared to the control.

Body weight (BW) was measured repeatedly throughout the individual stress experiments. The two-way ANOVA revealed significant interactions between stress types (EFSS, FSS, PSS) and exposure times (7, 14 and 21 days) [*F_(8,420)_ = *14.300, *p*<0.001] in the BW. There was a significant main effect of stress types [*F_(2,420)_ = *1286.413, *p*<0.001] and exposure times [*F_(4,420)_ = *812,974, *p*<0.001]. The rats subjected to EFSS gained significantly less BW than the control animals (Fig. 1A). EFSS caused a significant lack of gain in BW, but only in response to chronic stress [on the 14^th^ day [*F_(2,17)_ = *3.026, *p = *0.05] group 5: *p* = 0.049; on the 21^st^ day group 5: [*t_(10)_ = *10 , *p = *0.019 ] (Fig. 1A). However, exposure to FSS and PSS did not provoke any appreciable difference in BW (Fig. 1B,C).

**Figure 2:**
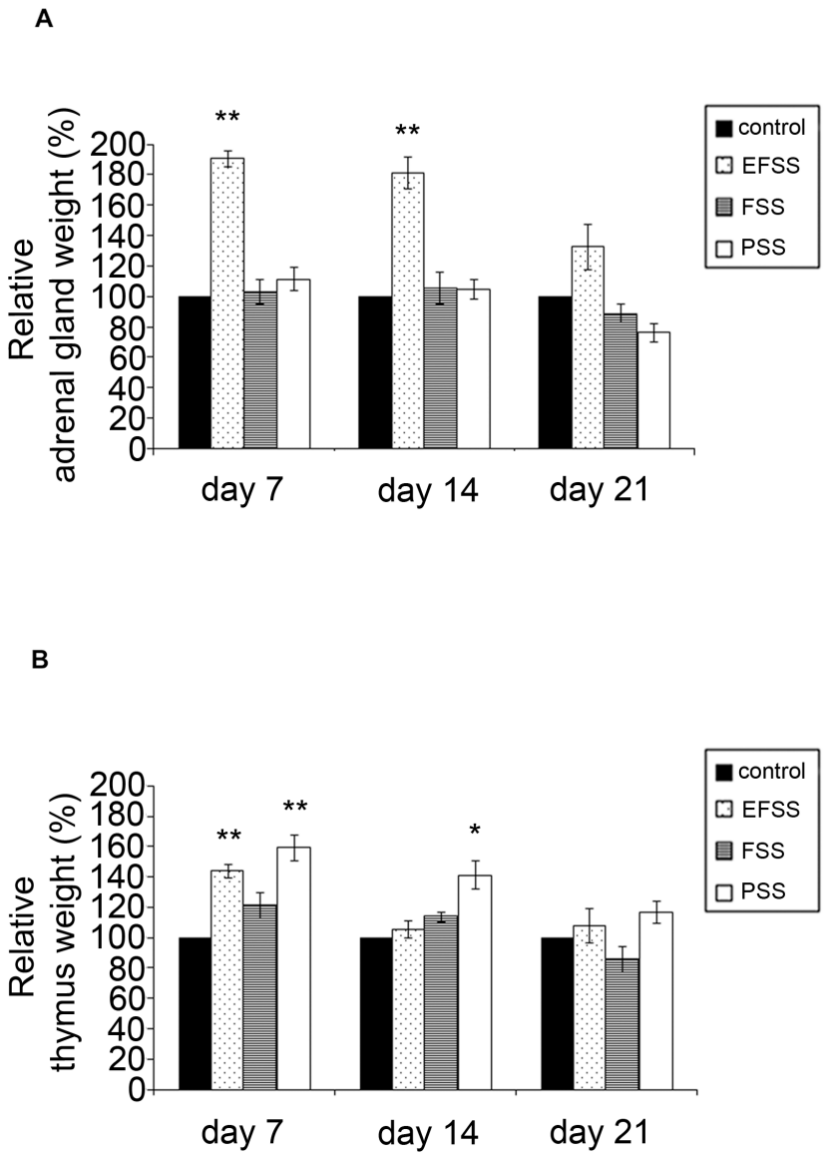
Stress type-dependent alterations of the weights of the adrenal glands and thymus. Effects of electric foot shock stress (EFSS), forced swimming stress (FSS) and psychosocial stress (PSS) on the weights of the adrenal glands (**A**) and the thymus (**B**) of rats, measured every 7 days. Results are expressed as percentages of the control (unstressed rats). Values for each group are means ± SEM, n = 6–10. *p<0.05 and **p<0.01: significant differences as compared to the control.

Figure 2A shows effects of EFSS, FSS and PSS on the weights of the adrenal glands of rats. The two-way ANOVA revealed significant interactions between stress types (EFSS, FSS, PSS) and exposure times (7, 14 and 21 days) [*F_(6,67)_ = *2.820, *p = *0.017] in the weights of the adrenal gland. There was a significant main effect of stress types [*F_(2,67)_ = *4.039, *p = *0.022] and exposure times [*F_(3,67)_ = *11.037, *p*<0.001]. The adrenal gland weight relative to the BW was significantly elevated by EFSS (Fig. 2A). EFSS induced a significant increase [*F_(3,19)_ = *13.657, *p*<0.001] within 7 days [*p*<0.001] (Fig. 2A); the adrenal gland weight was also increased on day 14 [*p*<0.001], but there was no significant elevation on day 21 (Fig. 2A). In contrast, FSS and PSS did not result in any significant change in the weight of the adrenal gland (Fig. 2A).

Figure 2B shows effects of EFSS, FSS and PSS on the weights of the thymus of rats. The two-way ANOVA did not reveal significant interactions between stress types (EFSS, FSS, PSS) and exposure times (7, 14 and 21 days) [*F_(6,67)_ = *1.165, *p = *0.335] in the weights of the thymus. There was a significant main effect of stress types [*F_(2,67)_ = *10.714, *p<*0.001] and exposure times [*F_(3,67)_ = *12.230, *p*<0.001]. EFSS, FSS and PSS caused a transient increase in the thymus weight relative to BW, after which a decreasing tendency was detected (Fig. 2B).

### Expressions of β-actin, cofilin and MAPK-1 mRNA in the rat brain after different stressful stimuli

**Figure 3:**
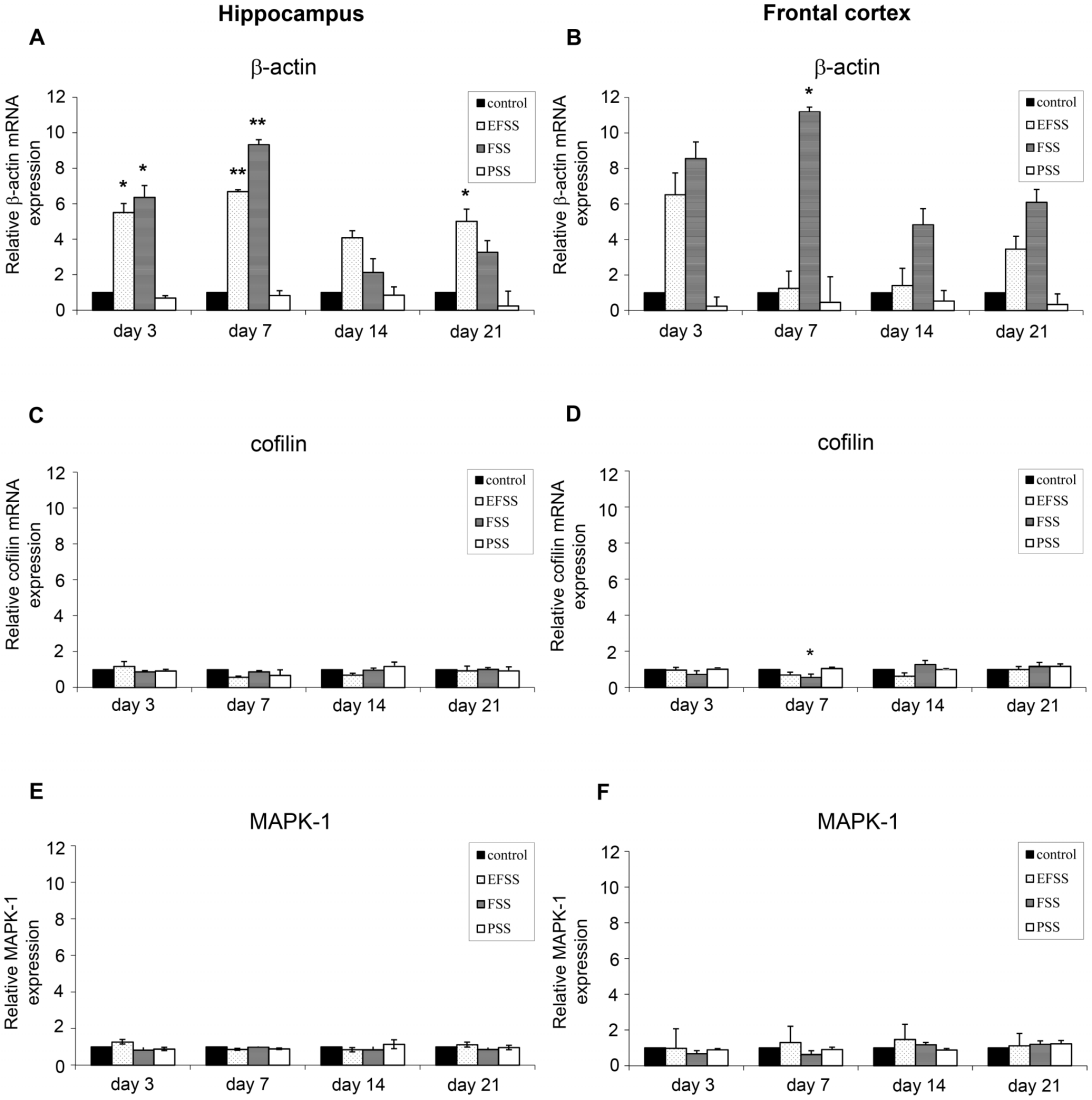
Stress type-dependent transcriptional alterations. Effects of electric foot shock stress (EFSS), forced swimming stress (FSS) and psychosocial stress (PSS) on the expressions of β-actin (**A, B**), cofilin (**C, D**) and MAPK-1 (**E, F**) mRNA in the rat hippocampus and frontal cortex. GAPDH was used as reference gene. Values for each group are means ± SEM, n = 6–10. *p<0.05 and **p<0.01: significant differences as compared to the control.

Figures 3A–F show the expression of β-actin (A,B), cofilin (C,D) and MAPK-1 (E,F) mRNA in the rat hippocampus and frontal cortex. The two-way ANOVA revealed significant interactions between stress types (EFSS, FSS, PSS) and exposure times (3, 7, 14 and 21 days) in the β-actin mRNA expression in the hippocampus [*F_(8,76)_* = 3.64, *p = *0.01]. There was a significant main effect of stress types [*F_(2,76)_ = *43.159, *p<*0.001] and exposure times [*F_(4,76)_ = *5.781, *p*<0.001]. In the hippocampus, EFSS [*F_(5,30)_ = *4.663, *p = *0.003] and FSS [*F_(4,19)_ = *4.510, *p = *0.01] caused significant increases in β-actin mRNA expression by day 3 [EFSS *p* = 0.034 and FSS *p = *0.05] (Fig. 3A). A biphasic U-shaped time course was detected in the case of EFSS. The β-actin mRNA level was found to be elevated on days 3, 7 and 21, respectively, but not on day 14 (Fig. 3A).

In the case of FSS, the time course was not U-shaped: significant elevations were observed on days 3 and 7, but there were no changes at the later time points (Fig. 3A). In contrast to the physical stressors, PSS did not influence the β-actin mRNA transcription in the hippocampus or the frontal cortex (Fig. 3A,B).

The two-way ANOVA revealed significant interactions between stress types (EFSS, FSS, PSS) and exposure times (3, 7, 14 and 21 days) in the β-actin mRNA expression in the frontal cortex [*F_(8,82)_* = 2.788, *p = *0.009]. There was a significant main effect of stress type [*F_(2,82)_ = *13.524; *p*<0.001], but the two-way ANOVA did not reveal a significant main effect of exposure times (3, 7, 14 and 21 days) in the β-actin mRNA expression in the frontal cortex [ *F_(2,82)_ = *1.113; *p = 0.356*]. FSS [*F_(4,28)_ = *7.266, *p = *0.001] caused significant increases in β-actin mRNA expression by day 7 [*p* = 0.032] (Fig. 3B).

The two-way ANOVA did not reveal significant interactions between stress types (EFSS, FSS, PSS) and exposure times (3, 7, 14 and 21 days) in the cofilin mRNA expression in the hippocampus [*F_(8,87)_* = 1.756, *p = *0.097]. EFSS, FSS and PSS had no effect on the expression of cofilin mRNA at any tested time point (Fig. 3C).

The two-way ANOVA revealed significant interactions between stress types (EFSS, FSS, PSS) and exposure times (3, 7, 14 and 21 days) in the cofilin mRNA expression in the cortex [*F_(8,85)_* = 3.885, *p = *0.01]. There was a significant main effect of stress types [*F_(2,85)_ = *712.122, *p<*0.001] and exposure times [*F_(4,85)_ = *6.461, *p<*0.001]. FSS decreased the cofilin mRNA expression [*F_(4,24)_ = *7.266, *p<*0.001] significantly by day 7 [*p = *0.032] (Fig. 3D).

The two-way ANOVA did not reveal significant interactions between stress types (EFSS, FSS, PSS) and exposure times (3, 7, 14 and 21 days) in the MAPK-1 mRNA expression in the hippocampus [*F_(8,84)_* = 2.011, *p = *0.055] or in the frontal cortex [*F_(8,84)_* = 0.463, *p = *0.962] (Fig. 3E,F). EFSS, FSS and PSS had no effect on the expression of MAPK-1 mRNA at any tested time point (Fig. 3E,F).

### Levels of β-actin and cofilin protein in the rat brain after different stressful stimuli

**Figure 4:**
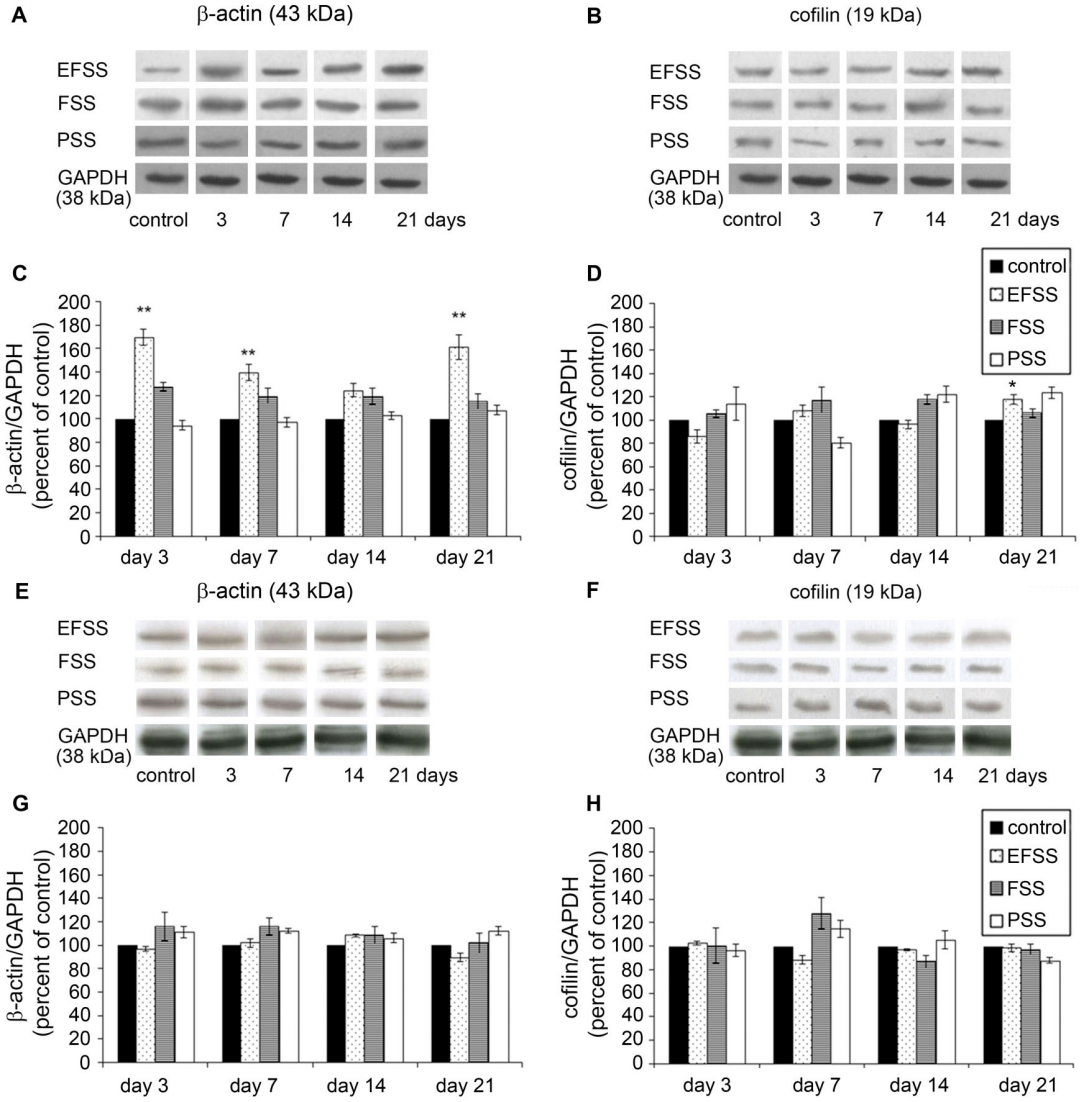
Western blot analysis of β-actin and cofilin after different stressors in hippocampus and frontal cortex. The specific bands for β-actin and cofilin in rat hippocampus (**A, B**) and frontal cortex (**E, F**) appeared at 43 kDa and 19 kDa, respectively. Antibodies used are described in Materials and methods. Semi-quantitative representations of electric foot shock stress (EFSS), forced swimming stress (FSS) and psychosocial stress (PSS) on the levels of β-actin and cofilin protein in the rat hippocampus (**C, D**) and frontal cortex (**G, H**). Results are expressed as percentages of the control (unstressed rats). GAPDH (38 kDa) was used as reference gene. Values for each group are means ± SEM, n = 6–10. *p<0.05 and **p<0.01: significant differences as compared to the control.

Figure 4 depicts representative β-actin or cofilin immunoblots after different types of stress. The β-actin or cofilin signals of the homogenates from the hippocampus were resolved at approximately 43 kDa, and 19 kDa, respectively (Fig. 4).

Figure 4 A–D shows the changes of β-actin and cofilin protein induced by the different stress types in the rat hippocampus. The two-way ANOVA revealed significant interactions between stress types (EFSS, FSS, PSS) and exposure times (3, 7, 14 and 21 days) in the levels of β-actin in the hippocampus [*F_(8,75)_* = 4.196, *p<*0.001]. There was a significant main effect of stress types [*F _(2,75)_ = *45.983, *p<*0.001] and exposure times [*F_(4,75)_ = *8.886, *p<*0.001]. Western blot experiments revealed statistically significant elevations in the hippocampal β-actin levels of exposure to EFSS [[*F _(4,25)_ = *13.288, *p<*0.001] day 3: *p*<0.001; day 7: *p = *0.012]; then, following a transient reduction on day 14, the β-actin protein level was again significantly increased by day 21 of exposure to EFSS [*p*<0.001] (Fig. 4C). Thus, similarly to the changes induced by EFSS in the transcription of β-actin mRNA, the protein level changes described a U-shaped time course (Fig. 3A, 4C). Neither FSS nor PSS modified the hippocampal β-actin levels significantly (Fig. 4C).

The two-way ANOVA revealed significant interactions between stress types (EFSS, FSS, PSS) and exposure times (3, 7, 14 and 21 days) in the levels of cofilin in the hippocampus [*F_(8,75)_* = 4.945, *p<*0.001]. There was a significant main effect of stress types [*F_(2,75)_ = *99.013, *p<*0.001] and exposure times [*F_(4,75)_ = *4.665, *p = *0.02]. The hippocampal cofilin level increased only in the EFSS-exposed group [*F _(4,25)_ = *8.065, *p<*0.001], where the elevation was significant by day 21 [*p = *0.026] (Fig. 4D).

In the frontal cortex, none of the applied stressors caused any significant changes in the level of either β-actin or cofilin (Fig. 4G,H).

## Discussion

The main finding of the present study was that the quantitative pattern of cytoskeletal stress response in the rat brain is unique to the stress model used to trigger it. The various stress models employed in this study affected the mRNA expression and protein levels of β-actin and cofilin differently; of the various physical and psychosocial stressors, EFSS induced the most pronounced changes in the investigated cytoskeletal markers. Another important observation was that the effects of this type of stress were region-specific, since changes were detected only in the hippocampus.

Our present results revealed that the levels of both β-actin mRNA and protein underwent biphasic dynamic changes in response to EFSS during the examined 3-week period. Previously, we demonstrated that RS induced biphasic dynamic changes in the transcription and protein translation of the main cytoskeletal component, β-actin, in an *in vivo* rat model, selectively in the hippocampal region [15]. The stress changes in β-actin transcription show a somewhat different pattern after EFSS compared to RS [15]: the initial elevation of the β-actin mRNA level was longer-lasting in the case of EFSS. However, these slower kinetic properties were not reflected in the protein level. In the first week of the stress period, the amount of β-actin in the hippocampus increased, then normalized and subsequently increased again. A possible explanation for these kinetic characteristics may be that short periods of stress serve an adaptive function, while longer durations may result in more profound changes through the depletion of compensatory mechanisms.

Examining the stress induced changes of regulating proteins cofilin and MAPK-1 of the actin filament dynamics, our study interestingly indicated that neither cofilin nor MAPK-1 were altered when the type of stressors, brain regions and time points were compared, in contrast to our previous work [15]. While the β-actin level pattern after EFSS was similar to earlier described stress type-dependent changes [15], the alteration in cofilin and MAPK-1 transcription and translation in response to EFSS differed considerably. The biphasic time course of mRNA levels of regulating factors and the elevation in cofilin protein levels were not demonstrated after EFSS. Results from a previous study from our group [15] and these newest data suggest that the changes induced in β-actin transcription and translation by RS and EFSS may be differently regulated. RS, but not EFSS may modify the actin dynamics (actin filament assembly/disassembly) and stabilization through regulation of the actin-depolymerizing factor/cofilin family and MAPK-1 [15].

Results from a previous study from our group [15] and these newest data suggest that the changes induced in β-actin transcription and translation by RS and EFSS may be consequences of different regulatory mechanisms. RS may modify the actin dynamics (actin filament assembly/disassembly) and stabilization through regulation of the actin-depolymerizing factor/cofilin family and MAPK-1 [15], whereas EFSS is not likely to have the same effect. Further investigations are necessary to clarify the roles of other regulator proteins, such as different kinases or drebrin in stress-induced cytoskeletal changes.

The elevation in the examined genes and proteins showed that the effects of acute or chronic EFSS are region-specific. The hippocampal cytoskeletal changes have been detected not only in this stress type, but similar changes were observed in a previous study after RS [15]. Recently, a morphological examination performed on cultured rat hippocampal slices demonstrated that glucocorticoid engaged the cofilin signaling pathway involved in regulating actin polymerization [13]. Since the major cytoskeletal component of the dendritic spines is filamentous actin, the local actin dynamics determine the changes in spine shape, numbers and size [7]. Previous reports demonstrated that chronic stress induces dendritic atrophy of the hippocampal pyramidal neurons and reduces the number of hippocampal neurons [23], [39]. Our findings are in agreement with these results and confirm that the hippocampus is one of the most stress-sensitive regions in the brain.

Besides EFSS, FSS and PSS are other commonly used animal models of depression or work-related stress [24], [32]. In the case of FSS and PSS the BW gain and the typical stress-related changes of adrenal gland weight or thymus weight were not observed. Additionally, FSS caused only a transient elevation of the β-actin mRNA expression only in the initial stages of the experiment in the hippocampal and the frontal cortex which did not induce any increase in protein levels. Our observations also indicate that neither acute nor chronic PSS caused any significant alterations in the investigated markers. These results suggest that the cytoskeletal changes are less sensitive to FSS or PSS, contrary to the biphasic effects induced by EFSS and RS [15].

The master regulator of the actin cytoskeleton expression, including the level of β-actin and cofilin, is a nuclear transcription factor, serum response factor (SRF) [40]. SRF activity is regulated by its co-factors, like myocardin-related transcription factors (MRTFs) [41]. The actin cytoskeleton is both an upstream regulator of MRTFs activity, with monomeric actin directly acting as a signal transducer, and a downstream effector, because of the many cytoskeletal target genes. In a conditional forebrain-specific SRF knockout mouse model shorter neuritic length and alteration of the cytoskeleton dynamics, impairments of growth cones dynamics and downregulation of actin mRNA levels in hippocampal neurons were observed [42], indicating the importance of actin levels in neuronal functions. A recent study identified SRF as a novel upstream mediator of FosB in nucleus accumbens after chronic social defeat stress, and implicated SRF in the development of depressive- and anxiety-like behaviors [43]. Based on these observations we hypothesize that the SRF/MRTFs signaling pathway may be responsible for the stress-induced β-actin changes in the hippocampus seen in our study.

Our experiment has important limitations. First, to confirm the physiological efficacy of the stress procedures, we measured only the body, adrenal gland and thymus weights. Although these stress-markers are often used parameters which represent the impact of stress, adrenocorticotropic hormone and corticosterone levels would be more informative to prove the intensity of the stressors. Second, the use of multiple control groups in each time point was ignored due to ethical reasons. Since the number of animals would have been greatly elevated, we compared the different experimental subgroups to one control group only for each stressor.

In conclusion, our study is the first to demonstrate that the levels of cytoskeleton proteins β-actin and cofilin increased in the hippocampus and frontal cortex of rats in models of electric foot shock and forced swimming stress, but not in psychosocial stress. These results suggest that the different stress models give rise to different quantitative and kinetic changes in the transcription and translation of the main components of cytoskeletal organization. Our results have important implications regarding the need for the careful selection of different stress models and their methodological importance. The fact that these molecular alterations were detected mostly in the hippocampus tends to suggest that this brain area may be the most stress-sensitive formation in the central nervous system. These changes additionally indicate strong stress-dependent neuronal cytoskeletal regulation in the rat brain, and our results may therefore contribute to the selection of appropriate stress models in connection with certain stress-related human conditions.
